# Synthesis, Characterization, *In Vitro* and *In Vivo* Screening of Unsymmetrical Borole Complexes of 2-Hydroxy-N-Phenylbenzamide and its Derivatives

**DOI:** 10.1155/MBD.2000.7

**Published:** 2000

**Authors:** Taruna Pandey, R. V. Singh

**Affiliations:** Department of Chemistry University of Rajasthan Jaipur 302004 India

## Abstract

Biochemical aspects, synthesis and characterization of some boron complexes of 2-hydroxy-N-phenylbenzamide (HOPhBenz) and its semicarbazone (HOPhBenz.SCZH) and thiosemicarbazone (HOPhBenz.TSCZH) are described. These derivatives were prepared by the reactions of 2-isopropoxy-4-methyl-1,3,2-dioxaborolane, and 2-isopropoxy-4-methyl-1,3,2-dioxaborinane with 2-hydroxy-N-phenylbenzamide, 1-[(2-hydroxyphenyl)-1-N-phenylamino]hydrazinecarboxamide (HOPhBenz.SCZH), and 1-[(2-hydroxyphenyl)-1-N-phenylamino]hydrazinecarbothioamide(HOPhBenz.TSCZH) in a 1:1 molar ratio. In order to assess the increase of the inhibitor potency, (HOPhBenz), (HOPhBenz.SCZH), (HOPhBenz.TSCZH) and their boron complexes have been tested *in vitro* against a number of pathogenic fungi and bacteria at different concentrations and were found to possess remarkable fungicidal and bactericidal properties. The testicular sperm density, testicular sperm morphology, sperm motility, density of cauda epididymal spermatozoa and fertility in mating trials and biochemical parameters of reproductive organs are discussed.


### SYNTHESIS, CHARACTERIZATION, *IN VITRO* AND *IN VIVO* SCREENING OF UNSYMMETRICAL BOROLE COMPLEXES OF 2-HYDROXY-N-PHENYLBENZAMIDE AND ITS DERIVATIVES

Taruna Pandey and R. V. Singh*

Department of Chemistry, University of Rajasthan, Jaipur 302004, India.

### ABSTRACT

Biochemical aspects, synthesis and characterization of some boron complexes of 2-hydroxy-N-phenylbenzamide (HOPhBenz) and its semicarbazone (HOPhBenz.SCZH) and thiosemicarbazone (HOPhBenz.TSCZH) are described. These derivatives were prepared by the reactions of 2-isopropoxy-4-methyl-1,3,2-dioxaborolane, and 2-isopropoxy-4-methyl-1,3,2-dioxaborinane with 2-hydroxy-N-phenyl-benzamide, 1-[(2-hydroxyphenyl)-1-N-phenylamino]hydrazinecarboxamide (HOPhBenz.SCZH), and 1-[(2-hydroxyphenyl)-1-N-phenylamino]hydrazinecarbothioamide(HOPhBenz.TSCZH) in a 1:1 molar ratio. In order to assess the increase of the inhibitor potency, (HOPhBenz), (HOPhBenz.SCZH), (HOPhBenz. TSCZH) and their boron complexes have been tested *in vitro* against a number of pathogenic fungi and bacteria at different concentrations and were found to possess remarkable fungicidal and bactericidal properties. The testicular sperm density, testicular sperm morphology, sperm motility, density of cauda epididymal spermatozoa and fertility in mating trials and biochemical parameters of reproductive organs are discussed.

### INTRODUCTION

Boron chemistry has continued to provide the most novel developments in the field of coordination chemistry. Inorganic chemistry of boron is much more diverse and complex than that of any other element in the Periodic Table and this is due to the elucidation of tremendous range of bonding intractions. Several organoboranes find promising applications in the synthesis of insect pheromones[1]. Boron azomethine complexes of various ligands have been found to possess conspicuous biocidal activity[2]. Boron-nitrogen compounds act as antivirals, fungicides, pesticides and even traces in medicine[3]. Bicyclic boron compounds have cytotoxic activity[4-6] and are used for the treatment of certain brain tumors[7]. The borohydride complexes of benzothiazepinole derivatives were found to inhibit $P_{388}$ Leukemia cell in mice[8]. There is still a sufficient scope for studies on N and S/O donor systems regarding their biochemical significance. Recently, a variety of azomethine complexes have been synthesized[9-12]. Thiosemicarbazones[13,14] and semicarbazones[15,16] are biologically important nitrogen and sulfur/oxygen donor ligands. Keeping all these uses in mind we have synthesized, characterized and screened some unsymmetrical borole complexes, the results of which are discussed in the present paper.

### RESULTS AND DISCUSSION

Reactions of unsymmetrical boroles with HOPhBenz, HOPhBenz.SCZH and HOPhBenz.TSCZH have been carried out in a 1:1 molar ratio in dry benzene. These reactions proceed with the liberation of the isopropanol/benzene azeotrope as :

$$B(R)(OPr^i) + HOPhBenz \xrightarrow[1:1]{Benzene} B(R)(OPhBenz) + Pr^iOH$$

$$B(R)(OPr^i) + HOPhBenz.SCZH \xrightarrow[1:1]{Benzene} B(R)(OPhBenz.SCZH) + Pr^iOH$$

$$B(R)(OPr^i) + HOPhBenz.TSCZH \xrightarrow[1:1]{Benzene} B(R)(OPhBenz.TSCZH) + Pr^iOH$$

Where R = $C_3H_6O_2$, and $C_4H_8O_2$

These reactions are quite facile and could be completed in 10-12h of refluxing. The resulting complexes are soluble in methanol, chloroform and DMSO. These are monomeric and non-electrolytes. Spectral studies comprising UV, IR and NMR ($^1H$, $^{11}B$ and $^{13}C$) support the proposed structures of the compounds. The electronic spectra of HOPhBenz, HOPhBenz.SCZH, HOPhBenz.TSCZH and borole complexes were recorded in methanol. The electronic spectra of conjugated bases exhibit three bands at *ca* 230, 270 and 320 nm. The bands at *ca* 230 and 270 nm are possibily due to $\pi$-$\pi^*$ transition within the benzene ring and around 320 nm may be due to n-$\pi^*$ transitions of the azomethine group. In the borole complexes these bands remain unaltered. The nitrogen atom of the >C=N group does not take part in the coordination as evidenced by the appearance of this band in the same position.

*IR Spectra*

On the basis of IR spectral studies an intramolecular hydrogen bonded structure for HOPhBenz has been established. In the case of HOPhBenz.SCZH the coordination can takes place either through the -NH phenyl group or through oxygen of the >C=O group. The carbonyl streching frequency (1685 $cm^{-1}$) shifts towards the lower frequency side, indicating that coordination has taken place through the carbonyl oxygen. The presence of a benzene ring at the nitrogen atom makes it less available for the coordination to the boron ion. HOPhBenz behaves purely as a bidentate chelating agent. In the IR spectra of conjugated bases broad bands at 3400-3200 $cm^{-1}$ are assigned to νOH due to the phenolic OH. This band disappears in the case of complexes indicating the possible loss of proton on complexation and subsequent formation of B-O bond due to the presence of new band in the region of 1345-1360 $cm^{-1}$ frequencies, respectively.

*$^{1}H$ NMR Spectra*

The proton magnetic resonance spectral data of HOPhBenz, HOPhBenz.SCZH, HOPhBenz.TSCZH and their boron complexes have been recorded in DMSO-$d_6$. The following structural inferences have been drawn by compairing the spectra of conjugated bases with those of corresponding boron complexes(Table I).

**Table I : $^{1}H$ and $^{11}B$ NMR Data (δ,ppm/JHz) of Conjugated Bases and Boron Complexes**

| Compound | -NH-Ph | -OH | -NH | $NH_2$ | Aromatic Protons* | | | | $^{11}B$ |
|---|---|---|---|---|---|---|---|---|---|
| | | | | | 1 | 2 | 3 | 4 | |
| [HOPhBenz] | 10.56s | 12.08 bs | - | - | 7.36(d) (7.3Hz) | 8.08(dd) (7.3Hz) (7.5Hz) | 6.80(dd) (7.5Hz) (7.2Hz) | 8.40(d) 7.2(Hz) | - |
| [B($C_3H_6O_2$) (OPhBenz)] | 10.62s | - | - | - | 6.96(d) (7.2Hz) | 7.92(dd) (7.2Hz) (7.3Hz) | 6.86(dd) (7.3Hz) (7.4Hz) | 8.57(d) 7.4(Hz) | 21.53 |
| [B($C_4H_8O_2$) (OPhBenz)] | 10.66s | - | - | - | 7.20(d) (7.3Hz) | 7.44(dd) (7.3Hz) (7.7Hz) | 6.97(dd) (7.7Hz) (7.5Hz) | 8.63(d) (7.5Hz) | 26.82 |
| [HOPhBenz. SCZH] | 10.42s | 12.16 bs | 10.00s | 2.70bs | 7.04(d) (7.1Hz) | 6.96(dd) (7.1Hz) (7.4Hz) | 7.20(dd) (7.4Hz) (7.2Hz) | 8.24(d) (7.2Hz) | - |
| [B($C_3H_6O_2$)(OPh Benz.SCZH)] | 10.84s | - | 10.34s | 2.76bs | 7.12(d) (7.5Hz) | 6.81(dd) (7.5Hz) (7.2Hz) | 6.88(dd) (7.2Hz) (7.3Hz) | 8.42(d) (7.3Hz) | 19.68 |
| [B($C_4H_8O_2$)(OPh Benz.SCZH)] | 10.93s | - | 10.43s | 2.82bs | 7.20(d) (7.1Hz) | 6.93(dd) (7.1Hz) (7.9Hz) | 8.16(dd) (7.9Hz) (7.5Hz) | 8.58(d) (7.5Hz) | 17.24 |
| [HOPhBenz. TSCZH] | 10.60s | 12.24 bs | 10.32s | 3.20 bs | 6.88(d) (7.4Hz) | 6.80(dd) (7.4Hz) (7.3Hz) | 8.24(dd) (7.3Hz) (7.5Hz) | 8.32(d) (7.5Hz) | - |
| [B($C_3H_6O_2$)(OPh Benz.TSCZH)] | 10.79s | - | 10.63s | 3.24bs | 7.36(d) (7.2Hz) | 6.97(dd) (7.2Hz) (7.3Hz) | 8.40(dd) (7.3Hz) (7.4Hz) | 8.54(d) (7.4Hz) | 31.15 |
| [B($C_4H_8O_2$)(OPh Benz.TSCZH)] | 10.86s | - | 10.75s | 3.29bs | 6.96(d) (7.5Hz) | 6.74(dd) (7.5Hz) (7.8Hz) | 8.16(dd) (7.8Hz) (7.3Hz) | 8.64(d) (7.3Hz) | 35.23 |

The disappearance of the -OH proton signal in case of complexes indicates the deprotonation and complexation through the functional group. The signal due to the NH protons attached to the phenyl ring remain unchanged in the complexes. The disappearance of the NH resonance signals of HOPhBenz.SCZH and HOPhBenz.TSCZH in the case of the complexes provides evidence for the complexation through the functional group. The -$NH_2$ protons remain almost unchanged.

*$^{13}C$ NMR Spectra*

The $^{13}C$ NMR spectral data have been recorded for HOPhBenz, HOPhBenz.SCZH, HOPhBenz.TSCZH and their boron complexes. The shifts of the carbon attached to O and S indicates the involvement of these atoms in coordination (Table II).

**Table II : $^{13}C$ NMR Spectral Data (δ, ppm) of Conjugated Bases and Their Boron Complexes**

| Compound | >C=S | >C=O/ >C=N | Ar-OH/ Ar-O | Ph-NH | Aromatic Carbons |
|---|---|---|---|---|---|
| [HOPhBenz] | - | 166.40 | 160.10 | 138.68 | $C_1$ 134.89; $C_2$ 129.97; $C_3$ 129.35; $C_4$ 125.66; $C_5$ 122.58; $C_6$ 120.74; $C_7$ 118.89; $C_8$ 118.96 |
| [$B(C_3H_6O_2)$(OPhBenz)] | - | 165.85 | 156.89 | 139.12 | $C_1$ 135.05; $C_2$ 131.25; $C_3$ 129.87; $C_4$ 124.76; $C_5$ 124.17; $C_6$ 120.98; $C_7$ 119.62; $C_8$ 118.68 |
| [$B(C_4H_8O_2)$(OPhBenz)] | - | 165.39 | 155.93 | 139.28 | $C_1$ 134.90; $C_2$ 130.45; $C_3$ 130.23; $C_4$ 126.05; $C_5$ 124.53; $C_6$ 121.15; $C_7$ 118.75; $C_8$ 119.16 |
| [HOPhBenz.SCZH] | - | 165.78 | 157.70 | 137.23 | $C_1$ 133.22; $C_2$ 129.15; $C_3$ 128.18; $C_4$ 125.41; $C_5$ 121.22; $C_6$ 120.21; $C_7$ 116.53; $C_8$ 116.42 |
| [$B(C_3H_6O_2)$(OPhBenz.SCZH)] | - | 162.82 | 153.25 | 135.64 | $C_1$ 133.87; $C_2$ 131.05; $C_3$ 127.43; $C_4$ 125.79; $C_5$ 122.64; $C_6$ 122.85; $C_7$ 117.09; $C_8$ 115.57 |
| [$B(C_4H_8O_2)$(OPhBenz.SCZH)] | | 160.98 | 151.35 | 134.49 | $C_1$ 134.62; $C_2$ 131.64; $C_3$ 128.98 $C_4$ 126.38; $C_5$ 121.88; $C_6$ 121.56 $C_7$ 116.05; $C_8$ 115.42 |
| [HOPhBenz.TSCZH] | 179.52 | 168.34 | 160.86 | 138.99 | $C_1$ 130.18; $C_2$ 129.79; $C_3$ 129.75 $C_4$ 125.58; $C_5$ 122.64; $C_6$ 120.68 $C_7$ 119.99; $C_8$ 118.94 |
| [$B(C_3H_6O_2)$(OPhBenz.TSCZH)] | 167.47 | 166.45 | 153.72 | 137.76 | $C_1$ 130.89; $C_2$ 130.12; $C_3$ 129.51 $C_4$ 126.03; $C_5$ 123.15; $C_6$ 121.82 $C_7$ 118.73; $C_8$ 118.65 |
| [$B(C_4H_8O_2)$(OPhBenz.TSCZH)] | 166.55 | 162.67 | 151.56 | 137.46 | $C_1$ 131.18; $C_2$ 129.88; $C_3$ 130.23 $C_4$ 125.84; $C_5$ 124.33; $C_6$ 120.85 $C_7$ 119.32; $C_8$ 119.74 |

*X-Ray Powder Diffraction Spectra*

The X-ray powder diffraction studies of two representative complexes [$B(C_4H_8O_2)$(OPhBenz.)] and [$B(C_3H_6O_2)$(OPhBenz.TSCZH)] have also been carried out. The results, show that the compounds belong to the orthorhombic crystal systems. The interplaner spacing values (dA°) and the miller indices h,k,l and a, b, c are listed in Tables III and IV.

*$^{11}B$ NMR Spectra*

The $^{11}B$ Nuclear magnetic resonance were observed between δ17.24 and 35.23 ppm (Table I) and which clearly supports a tricoordinated environment around the boron atom.

On the basis of the spectral evidences, the boron derivatives have been assigned the following structure with tricoordinated boron atom.

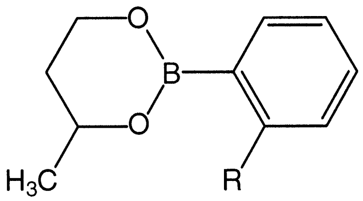

Where, R =

PhNHCO,
PhNHC:NN:C(OH)$NH_2$ and PhNHC:NN:C(SH)$NH_2$
n = 1 or 2

**Table III : X-Ray Powder Diffraction Data of [B($C_4H_8O_2$)(OPhBenz.)]**

| Peak No. | 2θ(deg.)(obs.) | d-spacing (obs.)(A°) | h | k | l |
|---|---|---|---|---|---|
| 1 | 7.8 | 14.2420 | 0 | 0 | 2 |
| 2 | 8.64 | 12.860 | 1 | 1 | 1 |
| 3 | 16.3 | 6.8330 | 0 | 2 | 3 |
| 4 | 17.75 | 6.2963 | 0 | 3 | 3 |
| 5 | 19.4 | 5.7492 | 0 | 0 | 5 |
| 6 | 21.2 | 5.2659 | 2 | 1 | 5 |
| 7 | 23.15 | 4.8297 | 0 | 0 | 6 |
| 8 | 23.45 | 4.7688 | 1 | 6 | 5 |
| 9 | 24.6 | 4.5471 | 3 | 0 | 5 |
| 10 | 25.4 | 4.4062 | 4 | 1 | 0 |
| 11 | 26.0 | 4.3062 | 2 | 7 | 4 |
| 12 | 27.15 | 4.1285 | 1 | 8 | 4 |
| 13 | 29.0 | 3.8688 | 0 | 6 | 8 |
| 14 | 32.4 | 3.4721 | 3 | 5 | 7 |
| 15 | 36.7 | 3.0769 | 1 | 8 | 7 |
| 16 | 37.3 | 3.0276 | 2 | 9 | 5 |
| 17 | 38.5 | 2.9381 | 3 | 7 | 6 |
| 18 | 43.5 | 2.6141 | 2 | 4 | 11 |

Refined value = a = 10.8256, b = 23.7350, c = 29.1565 (Orthorhombic system)

**Table IV : X-Ray Powder Diffraction Data of (B($C_3H_6O_2$)(OPhBenz.TSCZH)]**

| Peak No. | 2θ(deg.)(obs.) | d-spacing (obs.)(A°) | h | k | l |
|---|---|---|---|---|---|
| 1 | 8.54 | 13.0101 | 1 | 0 | 0 |
| 2 | 12.4 | 8.9693 | 1 | 3 | 1 |
| 3 | 18.6 | 5.9942 | 0 | 3 | 2 |
| 4 | 21.0 | 5.3155 | 0 | 5 | 5 |
| 5 | 22.0 | 5.0767 | 2 | 1 | 3 |
| 6 | 23.2 | 4.8174 | 1 | 1 | 4 |
| 7 | 24.7 | 4.5290 | 2 | 1 | 4 |
| 8 | 25.4 | 4.4062 | 1 | 5 | 3 |
| 9 | 26.2 | 4.2739 | 0 | 2 | 5 |
| 10 | 27.2 | 4.1195 | 0 | 5 | 4 |
| 11 | 28.0 | 4.0041 | 2 | 1 | 5 |
| 12 | 29.2 | 3.8429 | 4 | 4 | 1 |
| 13 | 31.6 | 3.5577 | 0 | 6 | 4 |
| 14 | 32.8 | 3.4309 | 1 | 5 | 5 |
| 15 | 33.0 | 3.4107 | 5 | 3 | 0 |
| 16 | 33.74 | 3.3380 | 3 | 8 | 0 |
| 17 | 34.8 | 3.2393 | 1 | 2 | 7 |
| 18 | 36.86 | 3.0688 | 5 | 5 | 2 |
| 19 | 37.4 | 3.0213 | 3 | 9 | 1 |
| 20 | 38.6 | 2.9308 | 0 | 9 | 4 |

Refined value = a = 13.0202 , b = 22.4347, c = 17.0650 (Orthorhombic system)

### *BIOCHEMICAL ASPECTS*

All the conjugated bases and their corresponding boron complexes were tested for the *in vitro* growth inhibitory activity against pathogenic fungi viz. *Fusarium oxysporum, Alternaria alternata, Rhizoctonia bataticola* and bacteria viz. *Staphylococcus aureus* and *Xanthomonas compestris*. Proper temperature, necessary nutrient and growth media free from other microorganism were employed for the preparation of cultures of fungi and bacteria using aseptic techniques. The antifungal activity of the complexes was evaluated by the Agar Plate Technique and antibacterial activity was evaluated by Paper Disc Plate Method[17]. The data of fungicidal and bactericidal activities of conjugated bases and their respective boron complexes against pathogenic fungi and bacteria were recorded in Tables V and VI.

**Table V : Fungicidal Screening Data of Conjugated Bases and Their Boron Complexes**

| Treatment (Compound) | Average Percentage Inhibition after 4 Days. | | | | | | | | | | | |
|---|---|---|---|---|---|---|---|---|---|---|---|---|
| | *Alternaria alternata* (Conc. in ppm) | | | | *Rhizoctoniabataticola* (Conc. in ppm) | | | | *Fusarium oxysporum* (Conc. in ppm) | | | |
| | 25 | 50 | 100 | 200 | 25 | 50 | 100 | 200 | 25 | 50 | 100 | 200 |
| [HOPhBenz] | 57 | 62 | 68 | 72 | 61 | 66 | 70 | 78 | 61 | 66 | 70 | 78 |
| [B($C_3H_6O_2$)(OPhBenz)] | 63 | 65 | 72 | 76 | 73 | 78 | 81 | 85 | 67 | 71 | 77 | 82 |
| [B($C_4H_8O_2$)(OPhBenz)] | 65 | 67 | 74 | 80 | 75 | 82 | 87 | 88 | 72 | 78 | 85 | 86 |
| [HOPhBenz.SCZH] | 62 | 64 | 74 | 80 | 62 | 68 | 72 | 80 | 62 | 68 | 72 | 80 |
| [B($C_3H_6O_2$)(OPhBenz.SCZH)] | 69 | 72 | 2 | 84 | 74 | 75 | 79 | 84 | 66 | 74 | 75 | 85 |
| [B($C_4H_8O_2$)(OPhBenz.SCZH)] | 72 | 78 | 85 | 86 | 77 | 79 | 82 | 86 | 69 | 77 | 80 | 88 |
| [HOPhBenz.TSCZH] | 67 | 72 | 81 | 85 | 65 | 70 | 76 | 80 | 65 | 70 | 76 | 80 |
| [B($C_3H_6O_2$)(OPhBenz.TSCZH)] | 73 | 78 | 84 | 87 | 72 | 78 | 83 | 86 | 72 | 77 | 79 | 84 |
| [B($C_4H_8O_2$)(OPhBenz.TSCZH)] | 77 | 82 | 86 | 88 | 75 | 82 | 87 | 88 | 76 | 80 | 82 | 86 |

**Table VI : Bactericidal Screening Data of Conjugated Bases and Their Boron Complexes**

| Treament (Compound) | *Staphylococcus aureus* | | *Xarthomonas compestris* | |
|---|---|---|---|---|
| | 500 ppm | 1000 ppm | 500 ppm | 1000 ppm |
| [HOPhBenz] | 6.0 | 7.0 | 3.0 | 4.0 |
| [B($C_3H_6O_2$)(OPhBenz)] | 8.0 | 9.5 | 4.0 | 6.0 |
| [B($C_4H_8O_2$)(OPhBenz)] | 8.5 | 10,0 | 5.5 | 7.5 |
| [HOPhBenz.SCZH] | 7.0 | 8.5 | 4.0 | 5.0 |
| [B($C_3H_6O_2$)(OPhBenz.SCZH)] | 10.0 | 9.0 | 6.5 | 7.5 |
| [B($C_4H_8O_2$)(OPhBenz.SCZH)] | 10.5 | 10.5 | 7.0 | 9.0 |
| [HOPhBenz.TSCZH] | 8.0 | 9.0 | 4.5 | 6.0 |
| [B($C_3H_6O_2$)(OPhBenz.TSCZH)] | 9.5 | 11.0 | 6.0 | 7.5 |
| [B($C_4H_8O_2$)(OPhBenz.TSCZH)] | 10.0 | 11.5 | 7.0 | 9.0 |

### Percent Disease Incidence Technique

In this method compounds were tested in the field for controlling the disease caused by the casual organism. Two concentrations i.e. 100 and 200 ppm were used in different plates and observations were recorded.

$$\text{Percent Disease Incidence} = \frac{\text{Number of infected plants}}{\text{Total no. of Plants observed}} \text{X } 100$$

The fungus *Alternaria alternata* in brinjal plants was used for these studies (Tables VIIa, VIIb, VIIc).

### Hanging Drop Method

In this technique total number of spores, germinated spores, and number of ungerminated spores were counted. $LD_{50}$ values have been calculated to compare HOPhBenz, HOPhBenz.TSCZH and their boron complexes by ploting graphs between concentration of conjugated bases or their complexes with the number of germinated spores.

### Antifertility Activity

Fourty two male albino rats were used in present study. Rats having weight between 175 to 200 g were divided into seven groups of six animals each. Animals were housed in polypropylene cages measuring 12"x10"x8" under controlled environmental conditions and fed with rat feed pellets and free acess to water.

**Table VII (a) : Efficacy of HOPhBenz, HOPhBenzTSCZH and B($C_4H_8O_2$)(OPhBenzTSCZH) Against Leaf Spot of Brinzal Plant caused by *Alternaria alternata* by Percent Disease Incidence Method.**

| Compound | Concentration in ppm | % Inhibition (Replication) | | | Average Statistical Values |
|---|---|---|---|---|---|
| HOPhBenz | 100 | 25 | 17 | 15 | (24.57) |
| | 200 | 15 | 11 | 14 | (21.38) |
| HOPhBenzTSCZH | 100 | 17 | 19 | 12 | (23.45) |
| | 200 | 13 | 12 | 10 | (19.94) |
| [B($C_4H_8O_2$)(OPhBenzTSCZH)] | 100 | 12 | 13 | 11 | (17.50 |
| | 200 | 9 | 7 | 8 | (16.55) |
| Bavistin (0.2%) | - | 8 | 11 | 9 | (17.75) |
| Control (water spray) | - | 34 | 39 | 42 | (38.24) |

S.Em = 0.94
CD at 5%= 2.68

**Table VII (b) : Antifungal Screening Data of Conjugated Bases and Their Complexes by Spore Germination Method**

| Compound | Conc.in (ppm) | *Alternaria alternata* Total No. of Spores | *Alternaria alternata* No. of Germinated Spores | *Alternaria alternata* No. of Ungerminated Spores | *Helminthosporium gramineum* Total No. of Spores | *Helminthosporium gramineum* No. of Germinated Spores | *Helminthosporium gramineum* No. of Ungerminated Spores |
|---|---|---|---|---|---|---|---|
| [HOPhBenz] | 500 | 10 | 1 | 9 | 12 | 2 | 10 |
| | 250 | 10 | 3 | 7 | 12 | 4 | 8 |
| | 125 | 10 | 6 | 4 | 12 | 5 | 7 |
| | 62.5 | 10 | 7 | 3 | 12 | 7 | 5 |
| [HOPhBenz.TSCZH] | 500 | 8 | 2 | 6 | 10 | 1 | 9 |
| | 250 | 8 | 3 | 5 | 10 | 3 | 7 |
| | 125 | 8 | 5 | 3 | 10 | 4 | 6 |
| | 62.5 | 8 | 7 | 1 | 10 | 6 | 4 |
| [B($C_3H_6O_2$)(OPhBenz)] | 500 | 12 | 1 | 11 | 10 | 1 | 9 |
| | 250 | 12 | 3 | 9 | 10 | 2 | 8 |
| | 125 | 12 | 7 | 5 | 10 | 3 | 7 |
| | 62.5 | 12 | 9 | 3 | 10 | 6 | 4 |
| [B($C_4H_8O_2$) (OPhBenz)] | 500 | 8 | 1 | 7 | 12 | 2 | 10 |
| | 250 | 8 | 2 | 6 | 12 | 4 | 8 |
| | 125 | 8 | 3 | 5 | 12 | 6 | 6 |
| | 62.5 | 8 | 5 | 3 | 12 | 8 | 4 |
| [B($C_3H_6O_2$)(OPhBenz.TSCZH)] | 500 | 8 | 2 | 6 | 10 | 2 | 8 |
| | 250 | 8 | 4 | 4 | 10 | 5 | 5 |
| | 125 | 8 | 5 | 3 | 10 | 6 | 4 |
| | 62.5 | 8 | 6 | 2 | 10 | 7 | 3 |
| [B($C_4H_8O_2$)(OPhBenz.TSCZH)] | 500 | 8 | 1 | 7 | 12 | 2 | 10 |
| | 250 | 8 | 2 | 6 | 12 | 3 | 9 |
| | 125 | 8 | 5 | 3 | 12 | 5 | 7 |
| | 62.5 | 8 | 7 | 1 | 12 | 7 | 5 |

**Table VII (c) : Comparison of $LD_{50}$ Value**

| Compound | $LD_{50}$ Value in *Alternaria alternata* | $LD_{50}$ value in *Helminthosporium gramineum* |
|---|---|---|
| (HOPhBenz) | 160 ppm | 89 ppm |
| (HOPhBenz.TSCZH) | 172 ppm | 86 ppm |
| [B($C_3H_6O_2$)(OPhBenz)] | 152 ppm | 80 ppm |
| [B($C_4H_8O_2$)(OPhBenz)] | 145 ppm | 72 ppm |
| [B($C_3H_6O_2$)(OPhBenz.TSCZH)] | 162 ppm | 83 ppm |
| [B($C_4H_8O_2$)(OPhBenz.TSCZH)] | 156 ppm | 75 ppm |

The group A served as vehicle (olive oil) treated control. In the group B, starting material, i.e., HOPhBenz. 50 mg/kg b.wt. suspended in olive oil was given orally for a period of 60 days. The animals of groups C,D,E,F and G received the same amount of HOPhBenz.TSCZH, [B($C_3H_6O_2$)(OPhBenz.)], [B($C_3H_6O_2$)

(OPhBenz.TSCZH)],[B($C_4H_8O_2$)(OPhBenz)] and [B($C_4H_8O_2$)(OPhBenz.TSCZH)] compounds, respectively for the same period. These animals were screened for fertility test and autopsied for detail biochemical changes. Reproductive organs were excised, blotted free of blood, weighed and were frozen for biochemical estimations. The sperm motility and density of cauda epididymal spermatozoa, total cholestrol, total protein, sialic acid, fructose and acid phosphatase were determined by Standard Laboratory Techniques.

**Table VIII : Effects of HOPhBenz, HOPhBenzTSCZH and Their Boron Complexes on Body Weight of Organ Weights of Male Rats**

| Group | Treatment | Body Weight (g) Initial | Final | Testes | Epididymis | Seminal vesicle | Ventral prostate |
|---|---|---|---|---|---|---|---|
| A | Control | 180.0 ± 20.0 | 210.0 ± 15.0 | 120.0 ± 30.5 | 415.0 ± 25.0 | 330.0 ± 20.70 | 250.0 ± 32.0 |
| B | [HOPhBenz] | 185.0 ± 20.0 | 220.0 ± 15.0[c] | 850.0 ± 30.0[b] | 310.0 ± 20.0[b] | 250.0 ± 10.5[b] | 240.0 ± 30.70[c] |
| C | [HOPhBenz.TSCZH] | 175.0 ± 13.0 | 190.0 ± 10.0[c] | 810.0 ± 15.0[a] | 290.0 ± 5.0[b] | 210.0 ± 7.5[a] | 170.0 ± 7.8[c] |
| D | [B($C_3H_6O_2$)(OPhBenz)] | 190.0 ± 15.0 | 225.0 ± 17.0[c] | 750.0 ± 15.0[a] | 270.0 ± 12.0[a] | 215.0 ± 18.0[a] | 180.0 ± 5.9[a] |
| E | [B($C_3H_6O_2$)(OPhBenz.TSCZH)] | 186.0 ± 12.0 | 218.0 ± 15.0[c] | 650.0 ± 18.0 [b] | 220.0 ± 10.0[b] | 190.0 ± 8.0[b] | 150.0 ± 6.8[b] |
| F | [B($C_4H_8O_2$)(OPhBenz)] | 195.0 ± 20.0 | 230.0 ± 20.0[c] | 730.0 ± 18.0[a] | 250.0 ± 15.0[a] | 210.0 ± 9.0[a] | 168.0 ± 9.8[a] |
| G | [B($C_4H_8O_2$)(OPhBenz.TSCZH)] | 188.0 ± 15.0 | 222.0 ± 18.0[c] | 630.0± 20.0[b] | 210.0 ± 12.0 | 285.0 ± 10.0[b] | 140.0 ± 9.6[b] |

Values ± SE of Six determinations
a = $p \leq 0.05$ Group B compared with Group A
b = $p \leq 0.001$ Group C compared with Group B
c = p = NS Groups D and F compared with Group B
Groups E and G compared with Group C

**Table IX : Sperm Dynamics and Fertility Test after Treatment With HOPhBenz, HOPhBenz.TSCZH and Their Boron Complexes**

| Group | Treatment | Sperm Motility Cauda epididymis | Sperm Density (million/ml) Testes | Epididymis | Fertility Test % |
|---|---|---|---|---|---|
| A | Control | 80.0 ± 5.1 | 1.95 ± 0.10 | 55.5 ± 1.75 | 99% + ve |
| B | [HOPhBenz] | 55.0 ± 3.1[b] | 0.89 ± 0.11[b] | 37.5 ± 0.90[b] | 71% - ve |
| C | [HOPhBenz.TSCZH] | 40.0 ± 4.1[b] | 0.70 ± 0.10[b] | 30.0 ± 0.95[b] | 85% - ve |
| D | [B($C_3H_6O_2$)(OPhBenz)] | 45.0 ± 2.1[a] | 0.65 ± 0.12[b] | 28.0 ± 0.91[b] | 88% - ve |
| E | [B($C_3H_6O_2$)(OPhBenz.TSCZH)] | 35.0 ± 3.1[b] | 0.55 ± 0.10[b] | 26.0 ± 0.90[b] | 90% - ve |
| F | [B($C_4H_8O_2$)(OPhBenz)] | 44.0 ± 3.1[a] | 0.68 ± 0.15[b] | 27.0 ± 0.80[b] | 92% - ve |
| G | [B($C_4H_8O_2$)(OPhBenz.TSCZH)] | 29.0 ± 2.1[b] | 0.65 ± 0.12[b] | 25.0 ± 0.91[b] | 93% - ve |

Values means ± SE of Six determinations.
a = $p \leq 0.05$ Group B compared with Group A
b = $p \leq 0.001$ Group C compared with Group B
c = p = NS Groups D and F compared with Group B
Groups E and G compared with Group C

(i) **Body and Organ Weights** : Body weight of rats were not affected after HOPhBenz, HOPhBenz.TSCZH and their boron complexes administration, However, the weight of testes, epididymis, seminal vesicle and ventral prostate were significantly described (Table VIII).
(ii) **Fertility Test** : The slugish motile spermatazoa were unable to fertilize normal cyclic females. The test was 71 to 93% negative in rats treated with these compounds.
(iii) **Sperm Motility** : The sperm motility declined significantly after treatment with the compounds.

(iv) **Sperm Density** : The sperm density in testes and cauda epididymis were declined significantly after treatment (Table IX).

### EXPERIMENTAL

All the chemicals and solvents were dried and purified by standard methods. The reactions were carried out under anhydrous conditions.

### Preparation of Hydrazinecarboxamide and Hydrazinecarbothioamide

Hydrazinecarboxamide and Hydrazinecarbothioamide of 2-hydroxy-N-phenyl-benzamide were prepared by the condensation of (HOPhBenz) with semicarbazide hydrochloride (in presence of sodium acetate) and thiosemicarbazide in a 1:1 molar ratio in alcoholic medium. The mixture was refluxed for 2-3 hours. On cooling, crystals were separated out which were recrystallized in the same solvent and finally dried in vacuum. The physical properties of these moieties are as follows :

Conjugated bases, Colour and M.P. (°C).

2-Hydroxy-N-phenylbenzamide (HOPhBenz), Light brown, 132-134

1-[(2-Hydroxyphenyl)-1-N-phenylamino]hydrazinecarboxamide (HOPhBenz.SCZH), Off white, 109-110

1-[(2-Hydroxyphenyl)-1-N-phenylamino]hydrazinecarbothioamide(HOPhBenz.TSCZH),Light pink,123-125

### Preparation of the Complexes

The unimolar reactions of unsymmetrical boroles with 2-hydroxy-N-phenylbenzamide and its derivatives were carried out in dry benzene. The reaction mixture was refluxed for 10-12h on a fractionating column and the progress of the reaction was monitored by the liberation of azeotropic isopropanol/benzene. After the completion of the reaction, the excess of the solvent was distilled off and the product was dried in vacuo. It was repeatedly washed with dry cyclohexane and again dried for 3-4h. These complexes were recrystalized in (1:1) solution of benzene and ether. The important physical properties and analytical data are given in Table X.

**Table X : Physical Properties and Analytical Data of the Boron Complexes**

| Compound | Colour | M.P. (°C) | Analysis % Found (Calcd.) | | | | | Mol. Wt. Found (Calcd.) |
|---|---|---|---|---|---|---|---|---|
| | | | C | H | N | S | B | |
| $[B(C_3H_6O_2)(OPhBenz)]$ | Cream | 214-218 | 64.33 (64.62) | 5.23 (5.42) | 4.15 (4.71) | - | 3.50 (3.65) | 332 (297) |
| $[B(C_4H_8O_2)(OPhBenz)]$ | Cream | 220-224 | 65.42 (65.63) | 5.59 (5.83) | 4.13 (4.50) | - | 3.35 (3.47) | 345 (311) |
| $[B(C_3H_6O_2)(OPhBenzSCZH)]$ | Cream | 180-185 | 57.47 (57.64) | 5.21 (5.40) | 15.10 (15.81) | - | 3.01 (3.05) | 398 (354) |
| $[B(C_4H_8O_2)(OPhBenzSCZH)]$ | Cream | 202-205 | 58.32 (58.75) | 5.25 (5.74) | 14.88 (15.23) | - | 2.80 (2.93) | 405 (368) |
| $[B(C_3H_6O_2)(OPhBenzTSCZH)]$ | Light brown | 170-174 | 56.03 (56.26) | 5.11 (5.50) | 14.13 (14.58) | 7.99 (8.34) | 2.70 (2.81) | 425 (384) |
| $[B(C_4H_8O_2)(OPhBenzTSCZH)]$ | Brown | 161-164 | 55.01 (55.15) | 4.98 (5.17) | 14.85 (15.13) | 8.23 (8.66) | 2.83 (2.91) | 417 (370) |

### Analytical Methods and Physical Measurements

IR spectra were recorded as KBr pellets or Nujol Mulls on a FTIR Spectrophotometer, Model Megna IR 550. $^1H$ NMR spectra were recorded on Jeol FX 90Q Spectrometer in DMSO-$d_6$ using TMS as the internal standard at 89.55 MHz. $^{11}B$ NMR spectra were recorded on the same Spectrometer. The electronic spectra were recorded in methanol on a Pye Unicam SP8-100 Spectrometer. Boron(III) was estimated volumetrically as boric acid[18]. Carbon and hydrogen analyses were carried out at the Microanalytical Laboratory of the Department. Nitrogen and Sulfur[19] were determined by Kjeldahl's and Messenger's methods, respectively. Conductivity measurements in dry DMF were made by a Systronics Conductivity Bridge Type 305 and molecular weights were determined by the Rast Camphor method.

The XRD measurements were performed on Phillips X-ray diffractometer (model PW-1840) having Fe-K$\alpha$ target and operated at 30 KV and 40mA. The diffraction data were refined using a least-squares refinment computer programme to obtain structural parameters. The programme essentially uses the least squares method of fitting of $d_{hkl}$ values for the different reflections. The intensity of Fe-K$\alpha$ radiation diffracted from the powder specimen was detected by a solid state detector and recorded as function of $2\theta$, where $\theta$ is the angle of incidence.

**Biochemical Changes**

Total protein and sialic acid contents of testes, epididymis, ventral prostate and seminal vesicle were depleted significantly after treatment with starting materials and their boron complexes. The acid phosphatase levels of testes epididymis and ventral prostate were also reduced significantly. A significant decrease in seminal vascular fructose contents was also noticed whereas the testicular cholestrol contents was increased significantly after the treatment with various compounds (Tables XI and XII).

**Table XI : Effects of HOPhBenz, HOPhBenzTSCZH and Their Boron Complexes on Biochemical Parameters of Reproductive Organs of Male Rats**

| Group | Treatment | Total protein (mg/gm) | | | | Sialic acid (mg/gm) | | | |
|---|---|---|---|---|---|---|---|---|---|
| | | Testes | Epididymis | Ventral prostate | Seminal vesicle | Testes | Epididymis | Seminal Vesicle | Ventral prostate |
| A | Control | 225.0 ± 18.0 | 208.0 ± 20.0 | 245.0 ± 15.0 | 260.0 ± 17.0 | 7.80 ± 0.9 | 6.90 ± 0.8 | 6.75 ± 0.9 | 7.10 ± 0.5 |
| B | [HOPhBenz] | 165.0 ± 15.0$^b$ | 170.0 ± 10.0$^a$ | 195.0 ± 10.0$^b$ | 210.0 ± 16.8$^b$ | 5.1 ± 0.6$^b$ | 5.0 0.4$^b$ | 5.6 ± 0.3$^b$ | 5.3 ± 0.4$^b$ |
| C | [HOPhBenz.TSCZH] | 140.0 ± 15.0$^a$ | 150.0 ± 7.8$^a$ | 155.0 ± 6.5$^a$ | 160.0 ± 9.6$^b$ | 4.1 ± 0.8$^a$ | 4.0 ± 0.3$^a$ | 4.8 ± 0.3$^a$ | 4.6 ± 0.3$^a$ |
| D | [$B(C_3H_6O_2)(OPhBenz)$] | 120.5 ± 10.0$^a$ | 128.0 ± 9.0$^a$ | 150.0 ± 6.0$^a$ | 170.0 ± 10.0$^b$ | 4.0 ± 0.3$^a$ | 3.9 ± 0.4$^a$ | 4.5 ± 0.4$^a$ | 4.1 ± 0.5$^a$ |
| E | [$B(C_3H_6O_2)$(OPhBenz. TSCZH)] | 100.0 ± 12.0$^a$ | 110.0 ± 8.0$^a$ | 120.0 ± 7.5$^b$ | 102.0 ± 5.0$^b$ | 3.9 ± 0.2$^b$ | 2.9 ± 0.5$^b$ | 3.5 ± 0.4$^b$ | 3.2 ± 0.2$^b$ |
| F | [$B(C_4H_8O_2)(OPhBenz)$] | 125.7 ± 12.0$^a$ | 130.0 ± 10.0$^a$ | 145.0 ± 8.0$^a$ | 165.0 ± 8.0$^b$ | 4.2 ± 0.6$^a$ | 3.7 ± 0.6$^a$ | 4.3 ± 0.5$^a$ | 4.4 ± 0.2$^a$ |
| G | [$B(C_4H_8O_2)$(OPhBenz. TSCZH)] | 105.0 ± 10.3$^a$ | 112.0 ± 9.0$^a$ | 121.0 ± 9.7$^b$ | 108.0 ± 9.0$^b$ | 4.1 ± 0.8$^a$ | 3.0 ± 0.6$^a$ | 3.6 ± 0.5$^b$ | 3.1 ± 0.1$^b$ |

Values means ± SE of Six determinations.
a = $p \leq 0.05$ Group B compared with Group A
b = $p \leq 0.001$ Group C compared with Group B
c = p = NS Groups D and F compared with Group B
Groups E and G compared with Group C

**Inferences**

The present study revealed that administration of the starting materials, HOPhBenz and HOPhBenz.TSCZH and their boron complexes caused a significant reduction in the weights of testes and other sex accessory glands. The development and function of male sex accessory glands the prostate and seminal vesicle are a well documented androgen dependent process.[20] The reduction in the weights of these organs could be due to reduced androgen level [21]. Sperm motility is considered as an important parameters in evaluating the fertility potential. HOPhBenz, HOPhBenz.TSCZH and their boron complexes significantly reduced the fertility of male rats. Since a number of androgen sensitive parameters (protein, sialic acid, fructose, acid phosphatase and total cholestrol) in target organs were found to be altered by these compounds, it is expected that the structure and function of epididymis and other sex accessory organs are changed. Most of the activities of HOPhBenz.TSCZH, and its effect on fertility is due to the presence of thio group[22]. Earlier it has been reported that boron exposure results in infertility, oligespernia and decreased libido in occupationally exposed men and rats. Our finding further suggested that addition of the boron moiety to the starting materials HOPhBenz or HOPhBenz.TSCZH increased their activity, and the boron complexes of HOPhBenz.TSCZH being indeed more effective fertility inhibitors.

**REFERENCES**

1. S. Singh, R. Dhillon and J. Singh, *Indian J. Chem.*, **306**, (1991) 355.
2. L. Bhal, J.P. Tandon and S.K. Sinha, *Curr. Sci.*, **53**, (1984) 566.
3. S. Padhye and G.B. Kauffman, *Coord. Chem. Rev.*, **63**, (1985) 127.
4. R. Csuk, H. Honig and C. Ramanin, *Monatsch. Chem.*, **113**, (1982) 1025.
5. R. Csuk, N. Muller and H. Sterk, *Z. Naturforsch.* B., **40**, (1985) 987.

**Table XII : Effects of HOPhBenz, HOPhBenzTSCZH and Their Boron Complexes on Biochemical Parameters of Reproductive Organs of Male Rats**

| Group | Treatment | Acid phosphatase (mg/ip Liberated/hr/mg tissue) | | | Fructose (mg/gm) | Cholesterol (mg/gm) |
|---|---|---|---|---|---|---|
| | | Testes | Epididymis | Ventral prostate | Seminal vesicle | Testes |
| A | Control | 3.9 ± 0.30 | 5.45 ± 0.06 | 3.0 ± 0.07 | 445.0 ± 30.0 | 7.60 ± 0.50 |
| B | [HOPhBenz] | 2.5 ± 0.11[b] | 3.41 ± 0.04[b] | 2.0 ± 0.01[a] | 380.0 ± 15.0[a] | 8.50 ± 0.5[a] |
| C | [HOPhBenz.TSCZH] | 2.1 ± 0.003[a] | 2.20 ± 0.04[a] | 1.70 ± 0.001[a] | 320.0 ± 17.0[b] | 9.1 ± 0.2[a] |
| D | $[B(C_3H_6O_2)(OPhBenz)]$ | 2.0 ± 0.01[a] | 2.30 ± 0.05[a] | 1.68 ± 0.001[a] | 325.0 ± 15.0[b] | 9.7 ± 0.3[a] |
| E | $[B(C_3H_6O_2)(OPhBenz.TSCZH)]$ | 1.9 ± 0.01[a] | 1.8 ± 0.02[a] | 1.3 ± 0.001[a] | 280.0 ± 10.0[b] | 9.9 ± 0.4[a] |
| F | $[B(C_4H_8O_2)(OPhBenz)]$ | 2.2 ± 0.01[a] | 2.25 ± 0.03[a] | 1.59 ± 0.002[a] | 300.0 ± 18.0[b] | 9.8 ± 0.4[a] |
| G | $[B(C_4H_8O_2)(OPhBenz.TSCZH)]$ | 2.0 ± 0.01[a] | 1.9 ± 0.03[a] | 1.4 ± 0.001[a] | 295.0 ± 15.0[b] | 9.78 ± 0.3[a] |

Values means ± SE of Six determinations.

a = $p \leq 0.05$ Group B compared with Group A

b = $p \leq 0.001$ Group C compared with Group B

c = p = NS Groups D and F compared with Group B

Groups E and G compared with Group C

6. R. Csuk, H. Honig, H. Weidmann and H.K. Zimmerman, *Arch. Pharm.*, **317**, (1984) 336.
7. C.A. Perks, A.J. Mill and J.A.B. Gibson, *British J. Radiol.*, **61**, (1988) 1115.
8. Hamariyakuhin, *Kogyo Co. Ltd., Jpn. Kokai Tokko Koho, Jpn.* 59, 101, 498 (1984); C.A. : **101**, (1984) 171292.
9. S. Belwal, S.C. Joshi and R.V. Singh, *Main Group Met. Chem.*, **20**, (1997) 313.
10. N. Gupta and R.V. Singh, *Main Group Met. Chem.*, **20**, (1997) 67.
11. S. Belwal and R.V. Singh, *Appl. Organomet. Chem.*, **12**, (1998) 39.
12. T. Pandey and R.V. Singh, *Indian, J. Chem.*, **37A**, (1998) 648.
13. D. Singh and R.V. Singh, *J. Inorg. Biochem.*, **15**, (1993) 227.
14. T. Pandey, V.P. Singh and R.V. Singh, *Main Group Met. Chem.*, **21**, (1998) 185.
15. C. Saxena, R.V. Singh and S.C. Joshi, *Bull. Chem, Soc. Jpn.*, **67**, (1994) 1007.
16. A. Saxena and J.P. Tandon, *Polyhedron*, **3**, (1984) 681.
17. T. Pandey, V.P. Singh and R.V. Singh, *Main Group Met. Chem.* **22**, (1999) 315.
18. L.H. Thomas, *J. Chem. Soc.*, (1946) 820.
19. C. Saxena, N.C. Bhardwaj, D. Singh and R.V. Singh, *Synth. React. Inorg. Met.-Org.Chem.*, **23**, (1993) 1391.
20. E.R. Rawlines, *"Bentary's Text Book of Pharmaceuticals" 8th ed. Boilliere Tindall, London* (1977).
21. D.S. Coff., Antrogen action and sex accessory tissue : In : Knobil, E. Meil, J.D. (eds). *The physiology of reproduction, Raven Press, New York,* (1988) 1081-1119.
22. H.F.S. Huang, Li M.T. Hagen, S.V. Zhang and R.S. Irwin, *Androgen modulation of the messenger ribonuclic acid of retinoic acid receptors in the prostate, seminal vesicle and kidney in the rats, Endorinol* **138 (2)**, (1997) 553-559.

**Received: September 20, 1999 - Accepted: October 8, 1999 - Received in revised camera-ready format: November 9, 1999**

